# Succession of soil microbial community in a developing mid-channel bar: The role of environmental disturbance and plant community

**DOI:** 10.3389/fmicb.2022.970529

**Published:** 2022-08-17

**Authors:** Fei Ye, Yiguo Hong, Jiapeng Wu, Xuemei Yi, Huub J. M. Op den Camp, Selina Sterup Moore, Teofilo Vamerali, Yu Wang

**Affiliations:** ^1^Key Laboratory for Water Quality and Conservation of the Pearl River Delta, Ministry of Education, Institute of Environmental Research at Greater Bay Area, Guangzhou University, Guangzhou, China; ^2^Chongqing Institute of Green and Intelligent Technology, Chinese Academy of Sciences, Chongqing, China; ^3^Department of Microbiology, Radboud Institute for Biological and Environmental Sciences (RIBES), Radboud University Nijmegen, Nijmegen, Netherlands; ^4^Department of Agronomy, Food, Natural Resources, Animals and Environment (DAFNAE), University of Padua, Padua, Italy

**Keywords:** soil bacterial community, ecological succession, plant-microbe association, disturbance, interdomain ecological networks, mid-channel bar

## Abstract

Succession of microbial and plant communities is crucial for the development and the stability of soil ecological functions. The relative role of plant communities and environmental disturbance in shaping the microbial community in a newly established habitat remains unclear. In this study, a mid-channel bar (MCB) exposed to an environmental disturbance gradient in the Yangtze River was studied to explore the effects of such disturbance and plant community traits on the succession of the soil microbial community. Bulk and rhizospheric soils were collected from the MCB and classified according to their level of exposure to environmental disturbance: head, central and tail. These subsequently underwent high-throughput sequencing and interdomain ecological network (IDEN) analysis to identify and characterize the predominant microbial groups present in the soils at each disturbance level. Furthermore, at each site, the presence and distribution of the plant community was also noted. The present study demonstrated that both bulk soil nutrients and plant community exhibited significant spatial distribution dependent on the level of disturbance and this influenced the composition of the microbial community. In less eroded parts of the MCB, i.e., the central, nutrients accumulated, promoting growths of plants. This in turn encouraged a more diverse microbial community, dominated by the bacterial genus *Pseudarthrobacter*. Plant showed a stronger association with bulk soil microbial communities compared to rhizosphere soil microbial communities. Particularly, *Triarrhena sacchariflora* and *Hemarthria altissima*, present in sites of low disturbance, exhibiting a more extensive plant-microbe association. They thus played a key role in shaping the soil microbial community. In general, however, plant species did not directly determine the composition of the bacterial community, but instead altered the nutritive state of the soil to promote microbial growth. Such findings are of significant value for conservation practices of newly formed ecosystems, which requires an integrated understanding of the role of environmental disturbance and plants on soil microbial community assemblage.

## Introduction

Microorganisms, particularly those considered beneficial, play a central role in soil life by regulating nutrient cycling and decomposition of organic matter, reducing soil-borne plant diseases and enhancing plant production (Coban et al., 2022). The succession of a microbial community has been an ecological topic of interest for an extended period of time (Fierer et al., 2010; Frossard et al., 2013). The study of microbial community structures in ecological successions helps to elucidate functional features of microorganisms in various environments and thereby the dynamic mechanisms of succession (Fierer et al., 2010; Zhong et al., 2018).

Ecological succession is generally defined as the orderly and predictable manner, in which populations of a newly colonized habitat change through time. Hereunder, primary and secondary succession exists. The former refers to population changes on an uncolonized substrate, while the latter refers to changes occurring on a previously colonized substrate that has recently undergone severe disruption (Fierer et al., 2010). Both categories of succession have been thoroughly addressed in literatures from the perspective of microbial communities and differing environmental disturbances (Kuramae et al., 2010; Cline and Zak, 2015; Zhong et al., 2018; Chai et al., 2019; Guo et al., 2019; Qiang et al., 2021).

Disturbances that act upon a habitat to provoke succession act in many forms. Most of the existing studies, however, have focused on anthropogenic disturbances in the form of farming, grazing, and pollution (Fichtner et al., 2014; Montagna et al., 2018; Qiang et al., 2021). These significantly alter the soil abiotic properties, especially those closely related to microbial metabolism. In the succession process, an array of abiotic properties such as pH, the C:N ratio, soil acid phosphatase, soil moisture, etc., have been found to change the composition of the microbial community (Montagna et al., 2018; Qiang et al., 2021). Consequently, many studies have investigated how soil physico-chemical qualities change with succession. Succession of a soil microbial community in response to environmental changes affect biogeochemical processes by improving nutrient availability of damaged soil and sustaining soil fertility; features closely linked to microbial functions (Kuramae et al., 2010; Cline and Zak, 2015; Cong et al., 2015).

Furthermore, it is evident that plant-microbe associations in soil are essential components of ecological succession (Chabrerie et al., 2003; Mark et al., 2006). Various functional characteristics of plants can lead to different modifications of bacterial and fungal communities (Chai et al., 2019). Indeed, such communities have been reported to change as a direct result of certain plant features, even acting as indictors of plant health (Sayer et al., 2017). For example, plants use species-specific root exudates to attract specific rhizospheric bacteria to the soil immediately surrounding the plant (Huang et al., 2014). Similarly, in an Alpine succession gradient, the soil fungal community was found to be useful as a proxy for the plant community along a disturbance gradient (Montagna et al., 2018).

Nonetheless, it is the total soil biota that governs the plant soil environment in a mutually beneficial feedback cycle by controlling activities like nitrogen fixation and mineral conversion (Araya et al., 2017). Therefore, the community of both soil microbes and plants may follow a similar succession trend along an environmental gradient (Qiang et al., 2021). However, studies aiming to elucidate such trends remain inconsistent and controversial. Clear associations between soil bacterial and fungal communities, and plant traits such as foliar C:N ratio and leaf dry matter content have been observed (Sayer et al., 2017). Other studies, however, have reported that the diversity of plants and soil biology did not correlate with environmental conditions (Lanzén et al., 2016; Montagna et al., 2018; Cameron et al., 2019). These discrepancies are probably due to different ecological contexts of the study site and their difference in type and severity of environmental disturbance. As such, direct comparisons between studies become problematic.

Mid-channel-bars (MCB), also known as river islands (i.e., an elevated region surrounded by river water) (Wen et al., 2020), determine river channel stability (Lou et al., 2018) and ecosystem functions (e.g., biodiversity and habitat provisioning) (Tabacchi et al., 2009; Tonkin et al., 2018). MCBs frequently exhibit predictable geomorphic development in terms of size, form, height, and placement under natural river regimes due to longitudinal and temporal fluctuations in flow and sediment load (Hooke and Yorke, 2011). In the geomorphic development of an MCB, the newly formed area experiences more disturbance than the aged part thereby forming a succession gradient between them.

According to our previous observations of the experimental site, obvious ecological succession of the plant community is evident along the MCB (Sun et al., 2021). However, the distribution and composition of the microbial community along the MCB remains unclear. The current study thus aimed to elucidate the relative roles of the abiotic properties and the plant community of the MCB in shaping the microbial community. Here, multidisciplinary approaches including field survey, high-throughput sequencing and inter-domain data analysis were applied and integrated in order to characterize the plant and soil microbial communities along the environmental disturbance gradient naturally found on the MCB. We aimed to address the following hypotheses: (i) community structures of different kingdoms are correlated and they are organized in a manner reflecting a response to the disturbance gradient of the MCB; (ii) apart from soil abiotic properties, the plant community structure significantly influences changes in the composition of the microbial community.

## Materials and methods

### Study site

**FIGURE 1:**
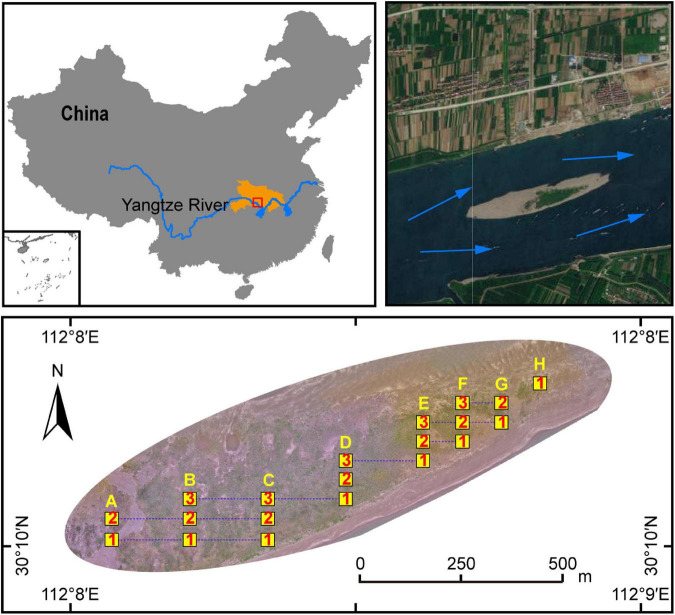
Schematic diagram of the study site and quadrats distribution in the Taipingkou mid-channel bar (MCB) (30°30′ N, 112°13′ E) located at the upper Jingjiang Reach of the Yangtze river. Blue arrows: direction of flow of the Yangtze River. Yellow squares: sampling quadrats. Numbers within yellow squares: the transect in which the quadrat is located. Capital letters above the yellow squares: the longitudinal position of the quadrats.

In order to explore the natural succession process of plants and associated soil microorganisms, an MCB named Taipingkou (30°30′ N, 112°13′ E) at the upper Jingjiang Reach of the Yangtze River was investigated in this research (Figure 1). The Taipingkou is a newly formed MCB which has been developing for nearly 50 years. It is mainly of sandy texture (Wang et al., 2015). The MCB experiences relatively little pollution input but is greatly affected by water flow regulations and sedimentation as a result of upstream dam operations. Historically, the head of the MCB is consistently collapsing with flow erosion, while a new tail is consistently formed due to sediment deposition (Wen et al., 2020). Therefore, the MCB tends to move downstream through time (Wang et al., 2015). The study area represents a humid subtropical monsoon climate with an annual mean temperature of 14.0−22.0°C. The annual precipitation is 1,100 mm characterized by great inter-annual variation due to monsoons.

### Quadrat set and sampling

A plant survey and sampling operation was conducted in May 2019. According to the dimensions of the MCB, 50 m × 50 m sample grids were setup each with 5 m × 5 m quadrats regularly distributed. They collectively formed a gradient-quadrat with a 25-m interval between each quadrat. Along the MCB, three transects with a total of 20 quadrats (8, 7, 5 quadrats for the transect 1, 2, and 3 respectively) were selected for plant survey and soil collection (Figure 1). The number of each plant species was recorded. The coverage (%) of each plant was calculated based on the vertical projection of exposed leaf area relative to the quadrat area. Five individuals of each plant species were randomly selected to measure species-specific height. The value of the importance of each species was calculated as:


I⁢m⁢p⁢o⁢r⁢t⁢a⁢n⁢c⁢e⁢v⁢a⁢l⁢u⁢e=(RD+RF+RC)/3

Where *RD* (relative height) was the density of one plant as a percentage of the total density of all plants in a quadrat; *RF* (relative frequency) was the frequency of one plant as a percentage of the frequency of all plants; *RC* (relative coverage) was the coverage of one plant as a percentage of the total plant coverage. In each quadrat, 1–2 plant species were identified as dominant plants based on their importance values.

**FIGURE 2:**
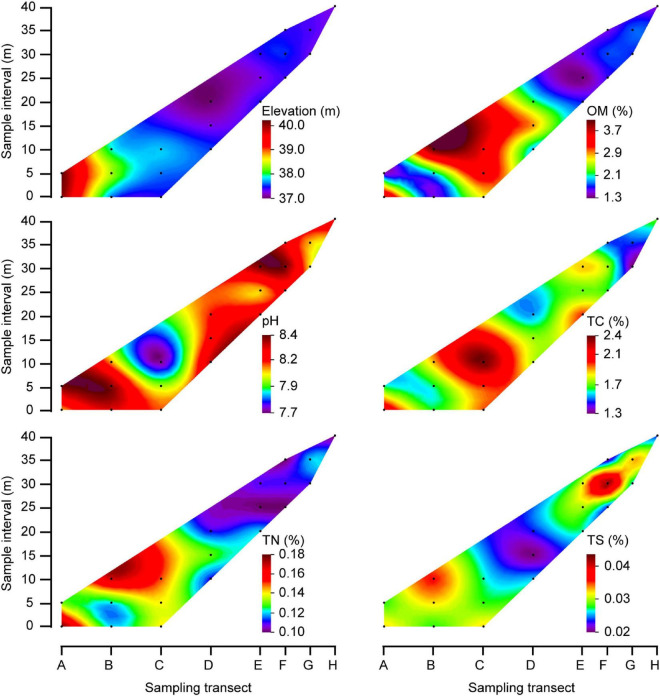
Contour plots showed physico-chemical properties of bulk soils in the MCB. The black dots indicate the sampling quadrats, which correspond to the quadrat orders depicted in Figure 1. The color fill plot represents a 3-dimensional bulk by plotting contours on a 2-dimensional format using Origin soft. Capital letters: longitudinal position of the quadrats.

**FIGURE 3:**
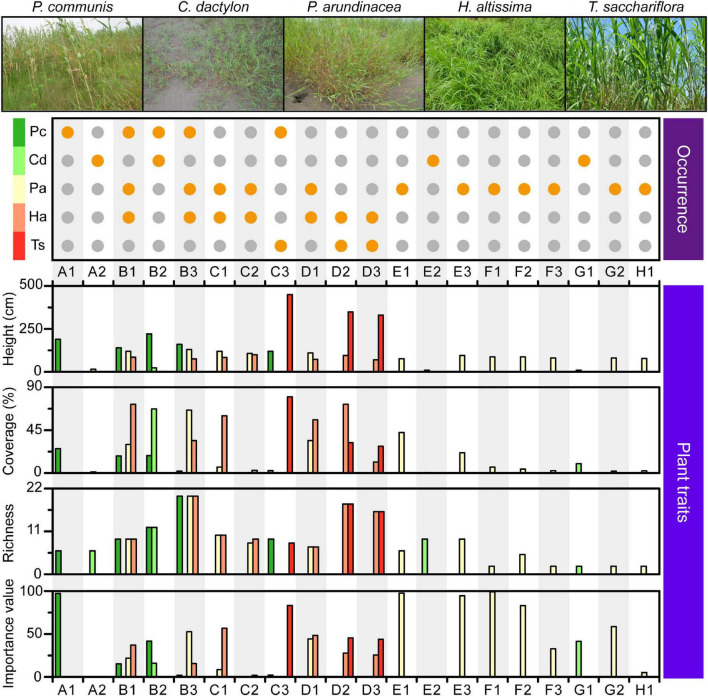
Characteristics of the plant community found within each quadrat. Orange dot in the top plot: occurrence of each plant species, denoted on the left, in the corresponding quadrat. Combination of capital letter and number: quadrat identity; capital letters and numbers: longitudinal position and transect order of quadrats, respectively. Pc, *P. communis*; Cd, *C. dactylon*; Pa, *P. arundinacea*; Ha, *H. altissima*; Ts, *T. sacchariflora*.

The sampling procedure of the rhizospheric soil was conducted as described in a previous study (Ye et al., 2021). In brief, the randomly selected plants were removed from each quadrat, and loosely adhering soil on roots was gently shaken off. Roots with closely adhering soil were put in saline phosphate buffer using sterilized forceps in order to obtain rhizospheric soil samples. Rhizospheric soils from the same plant species were mixed thoroughly to obtain a composite sample. In addition, five bulk soil samples (0−10 cm depth) from each of the 20 quadrats were collected using the five-point sampling method. These five samples were then mixed into one composite sample per quadrate. A total of 20 composite bulk soil samples and 31 composite rhizosphere soil samples were obtained. These samples were thereafter either stored at 4°C if later used for physico-chemical analysis, or at −20°C for DNA extraction. Based on the properties of the bulk soil (Figure 2) and the distribution of the plants (Figure 3), the quadrants were spatially divided into three groups: head (A1, A2, B1, B2), central (B3, C1, C2, C3, D1, D2, D3) and tail (E1, E2, E3, F1, F2, F3, G1, G2, H1) group.

### DNA extraction and sequencing

Roughly 0.25 g of soil from each sample was randomly selected to extract DNA using the DNeasy PowerSoil Kit (Qiagen, Hilden, Germany) following the protocol provided by the producer. The quantity and quality of extracted DNA was determined by 1% (w/v) agarose gel electrophoresis and a NanoDrop Lite Spectrophotometer (Thermo Scientific, Waltham, United States), respectively. The extracted DNA was stored at −80°C until later analysis.

The V3−V4 region of the bacterial 16S rRNA gene was amplified using primers 338F/806R (Lee et al., 2012). The polymerase chain reaction (PCR) condition, sequencing and data processing followed those published in our previous study (Ye et al., 2021). Briefly, the PCR products were purified with an AxyPre DNA Gel Extraction Kit (Axygen, Union City, United States), whereafter the purified amplicons were pooled in equimolar. The amplicons where sequenced through paired-end sequencing on an Illumina MiSeq PE300 platform (Majorbio Bio-Pharm Technology Co., Ltd., Shanghai, China).^1^ The bulk soils from quadrats of F2, F3, G2, and H1 were excluded from high-throughput sequencing analysis due to the failure of the PCR. Accordingly, rhizospheric soils from these four quadrats were also removed in the subsequent analysis.

Raw sequence data were demultiplexed and quality-filtered using Trimmomatic (v. 0.30) (Bolger et al., 2014). Operational taxonomic units (OTUs) were identified at 97% sequence similarity using Usearch (v. 7.0).^2^ The bacterial taxonomy was analyzed using the Ribosomal Database Project (RDP) with SILVA 132 database.^3^ OTUs with total sequences <20 in all samples were eliminated. To make samples comparable, sequences in each sample were rarefied to an even sequencing depth based on the sample with the lowest sequence reads, which was 22,850. Alpha diversity indices, including Shannon diversity, Chao1 richness, Heip evenness, and phylogenetic diversity, were calculated using Mothur (v. 1.30.1) based on the rarefied sequence data.

### Statistical analyses

To test the significant differences of soil physicochemical properties and alpha diversity of the bacterial community between bulk and rhizospheric soils, nonparametric Mann–Whitney *U* test was conducted using SPSS statistics 20.0 (IBM, Armonk, NY, United States). One-way analysis of variance (ANOVA) based on least significant difference (LSD) was used to test the significant differences of relative abundances of dominant bacterial phyla among different groups (SPSS statistics 20.0, IBM, Armonk, NY, United States). Principal coordinates analysis (PCoA) based on Bray-Curtis dissimilarity was conducted in CANOCO 5 software (Ter Braak and Šmilauer, 2012) on OTU level to demonstrate the distribution of the bacterial community in different samples. Analysis of similarity (ANOSIM) was further conducted to analyze the significant differences in the bacterial community among different groups by using vegan package in R v. 3.5.1 based on Bray-Curtis distances. Linear discriminant analysis effect size (LEfSe) was used to determine the differences in bacterial taxa among different sampling groups.^4^ The linear discriminant analysis (LDA) was used to estimate the effect of the abundance of each taxa on the differential effect (Ding et al., 2022). Higher LDA scores indicated significantly higher abundance.

Partial least squares path model (PLS-PM) was carried out to infer possible direct and indirect effects of flooding disturbance, plant community trait and soil conditions on bulk and rhizospheric soil bacterial communities. The flooding probability, which represented the level of flooding disturbance, was calculated as the percentage of times (days) each site was flooded in the past year until sampling day (Supplementary Figure 1). The PLS-PM was performed in R v. 4.1.1 with “plspm” package. The model path used 1,000 bootstraps to validate the estimates of path coefficients and the coefficients of determination (*R*^2^) (Cui et al., 2016). The observed variables with loading <0.7 were excluded in the PLS-PM construction. The model was assessed using the Goodness of Fit; a measure of the overall prediction performance. Indirect effects were calculated as the multiplied path coefficients between a predictor and a response variable.

### Interdomain ecological network analysis

Interdomain ecological networks (IDEN) were carried out to reveal the associations between plant and soil bacterial communities. The IDEN analysis processes was conducted using the interdomain ecological network analysis pipeline (IDENAP)^5^ on OTU level (Feng et al., 2019, 2022). To reduce type I errors, only OTUs with more than 20 sequences across the total samples were retained. The filtered datasets of microbial abundance and plant abundance (based on plant richness) were used to calculate the pairwise associations between plants and microbes using the sparse correlations for compositional data (SparCC) with default parameters. Here, 20 was included as the number of inference iterations, 10 for exclusion iterations, 0.1 as strength exclusion threshold, and 100 as the number of times shuffled. Two output files containing correlations and a pseudo *P* values matrix were exported (Friedman and Alm, 2012; Feng et al., 2019). Only the plant-microbe associations were retained for network analysis to allow focusing on interdomain species (plant and bacteria) associations. The detail analytical procedures can be found in the instructions of the IDENAP (see text footnote 5). The topological characterization of the constructed networks was analyzed to identify association patterns between plant and bacteria (Feng et al., 2019). The constructed networks were visualized using Gephi 0.9.1 (Bastian et al., 2009).

### Accession numbers

The raw sequencing data were deposited in the National Genomics Data Center (NGDC) Genome Sequence Archive (GSA)^6^ under accession number CRA004701.

## Results

### Physico-chemical properties of soil

The elevation of the sampling area ranged from 37.1 to 40.3 m above sea level, with elevations at the MCB head being higher than elevations at the tail (Figure 2). The organic matter (OM), total carbon (TC) and total nitrogen (TN) contents in bulk soils were 1.3−4.1%, 1.3−2.4%, and 0.10−0.18%, respectively. Although with some variation, these values were primarily higher in the central part of the MCB in respect to values found in either of the two ends (Figure 2). However, soil pH and total sulfur (TS) were lower in the central part than in either end, which was 7.7−8.4 and 0.02−0.04%, respectively. The OM (1.7−5.3%), TC (1.7−2.7%), TN (0.06−0.20%), and TS (0.02−0.07%) were significantly higher in rhizospheric soil than in bulk soil (Mann–Whitney *U* tests, *P* < 0.05) while pH (7.4−7.9) was significantly lower in rhizospheric soil (Mann–Whitney *U* tests, *P* < 0.001; Supplementary Table 1 and Supplementary Figure 2).

### Plant communities

A total of five dominant plant species were identified across the MCB, including the four perennial herbs *Phragmites communis*, *Cynodon dactylon*, *Hemarthria altissima*, *Triarrhena sacchariflora*, and the annual herb *Phalaris arundinacea* (Figure 3). *P. communis* was mainly found in the head of the MCB with height, coverage, richness and importance values ranging from 120 to 220 cm, 1.9−25.6%, 6−20, and 1.9−97.2, respectively (Figure 3 and Supplementary Table 2). *C. dactylon* was scattered across the head and tail of the MCB with height, coverage, richness and importance value ranging from 9 to 23 cm, 0.6−67.2%, 2−12, and 0.3−41.5, respectively. Unlike other plants, *P. arundinacea* was almost evenly distributed throughout the study area with height, coverage, richness and importance values ranging from 76 to 130 cm, 0.6−66.0%, 2−12, and 0.3−41.5, respectively. *H. altissima* was mainly observed in the head and central part, whereas *T. sacchariflora* tended to congregate in the central part (Figure 3). The height, coverage, richness and importance values of *H. altissima* and *T. sacchariflora* are depicted in Supplementary Table 2. It was noted that areas of high OM and TC were mostly dominated by *P. arundinacea* and *H. altissima*. Overall, different plant species exhibited distinct distribution patterns along the MCB, with the central part exhibiting a predominantly higher coverage and richness in the associated plant community.

### Soil bacterial community

**FIGURE 4:**
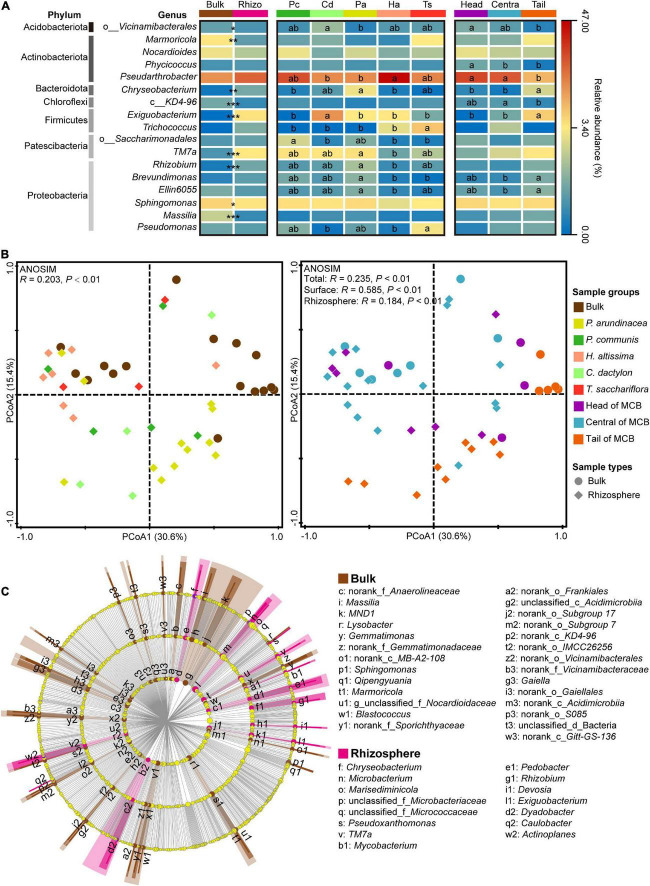
Bacterial communities in bulk and rhizospheric soils. **(A)** The abundance of dominant bacterial genera (relative abundance >1%) across different soil sample groups (bulk and rhizosphere, plant species, spatial parts). “*,” “**,” and “***” indicate the significant differences in the abundance of dominant bacterial genera between bulk and rhizospheric soils at *P* < 0.05, 0.01, and 0.001 based on the Mann-Whitney *U* test, respectively. Different lowercase letters in the heatmap indicate significant differences among different plant species and different spatial parts of the MCB based on a one-way ANOVA with LSD multiple comparisons at *P* < 0.05. **(B)** Principal coordinate analysis (PCoA) plot of the bacterial community based on Bray–Curtis distance on operational taxonomic unit (OTU) level. Analysis of similarity (ANOSIM) of bacterial community among different groups based on Bray–Curtis distance with 999 permutations. Left plot is species-specific color-coded while right plot is color-coded for MCB area. Round marks: bulk samples, marks of rhombus shape: rhizosphere samples. **(C)** Linear discriminant analysis effect size (LEfSe) of bacterial communities between bulk and rhizosphere soils. Bacteria with linear discriminant analysis (LDA) scores >3.0 are displayed in LEfSe cladogram. Circles in LEfSe indicate phylogenetic levels from order to genus, and only bacteria at genus level are labeled with taxonomy. The diameter of each circle is proportional to the abundance of the group. Rhizo, rhizosphere; Pc, *P. communis*; Cd, *C. dactylon*; Pa, *P. arundinacea*; Ha, *H. altissima*; Ts, *T. sacchariflora*.

A total of 1,659,408 high-quality sequences were obtained from the 47 soil samples. These were rarefied to obtain an even sequencing depth across all samples based on the sample with the lowest sequence reads of 22,850. Rarefied sequences were subsequently clustered into 3,383 operational taxonomic units (OTUs) at 97% similarity level. Among the 17 identified dominant bacterial genera (relative abundance >1%), the *Pseudarthrobacter* was the most abundant genus and accounted for 25.3% of all sequences, followed by *Sphingomonas* (5.9%), *Exiguobacterium* (3.4%), *TM7a* (3.0%), *Nocardioides* (2.8%), and *Marmoricola* (2.2%) (Figure 4A). The relative abundance of *Pseudarthrobacter* in rhizospheric soil (28.6%) was higher than that in bulk soil (20.0%). The rhizospheric soil of *H. altissima* harbored the most abundant fraction of *Pseudarthrobacter* (46.1%), which was significantly higher than that of the rhizospheric soil of *C. dactylon* (21.2%) and *P. arundinacea* (20.9%) (one-way ANOVA, *P* < 0.05; Figure 4A). Moreover, the relative abundance of *Pseudarthrobacter* in the head (34.0%) and central (30.7%) MCB were significantly higher than that in the tail (13.3%) (one-way ANOVA, *P* < 0.05; Figure 4A).

A distinct separation of bacterial communities between bulk and rhizospheric soils was evaluated by principal coordinates analysis (PCoA), and further confirmed by analysis of similarities (ANOSIM, *P* < 0.01; Figure 4B). In addition, the bacterial community of bulk (ANOSIM, *P* < 0.01) and rhizospheric soil (ANOSIM, *P* < 0.01) was significantly divided by the three spatial groups of the MCB (ANOSIM, *P* < 0.01; Figure 4B). To identify the bacterial taxa most indicative of different sampling groups, and thus of various disturbance levels, a linear discriminant analysis effect size (LEfSe) analysis was performed on the bacterial community from order to genus level. More bacterial taxa were identified in bulk soil (27 genera, 22 families, and 20 orders) than in rhizospheric soils (15 genera, 10 families, and 7 orders) (Figure 4C). *Marmoricola* was the most abundant genus in bulk soil, followed by *Sphingomonas* and *Massilia*, whereas the rhizospheric soil had a higher abundance of *Exiguobacterium*, *TM7a*, and *Chryseobacterium* (Supplementary Figure 3). For the bacterial community in soil across the three spatial groups of the MCB, only 1 and 4 taxa were highly represented in the head and central zones respectively, while no taxon was overrepresented in the tail soil (Supplementary Figure 3). The family of *Roseiflexaceae*, and the order of *Clostridiales* were the most abundant bacterial taxa in head and central soils, respectively.

### Alpha diversity of bacterial community

**FIGURE 5:**
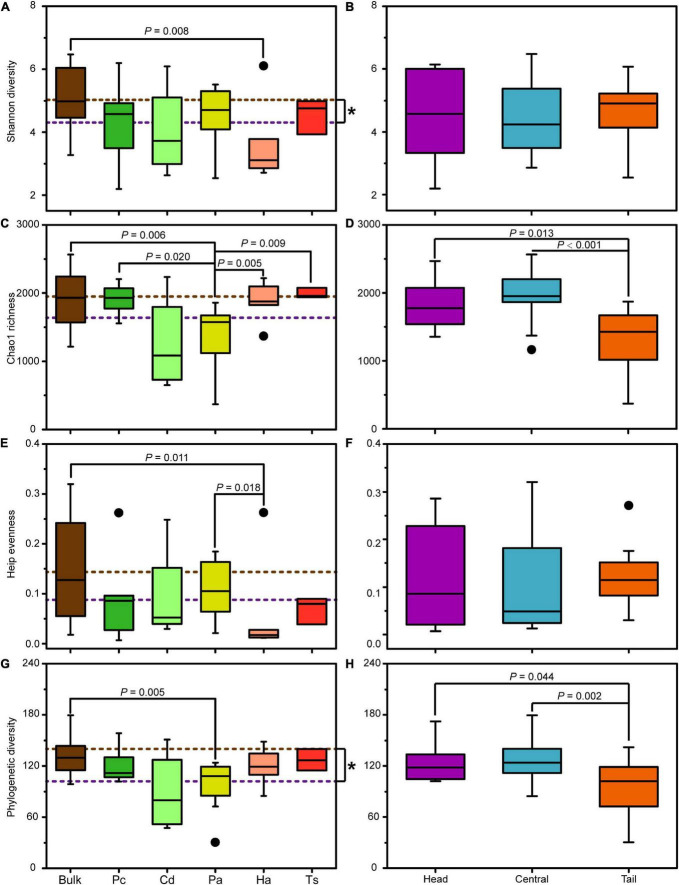
Alpha diversity indices of bacterial communities in bulk and rhizospheric soils **(A,C,E,G)**, and in bulk soils from the head, central, and tail part of the MCB **(B,D,F,H)**. Pc, *P. communis*; Cd, *C. dactylon*; Pa, *P. arundinacea*; Ha, *H. altissima*; Ts, *T. sacchariflora*. The brown and purple dotted lines: the mean value of community diversity in bulk and rhizospheric soils, respectively. The *P* values with significant differences at 0.05 level based on nonparametric Mann–Whitney *U* tests are indicated above each box plot. “*” indicates the significant difference at 0.05 level based on nonparametric Mann–Whitney *U* tests.

In general, the alpha diversity indices of the bacterial community in bulk soil were higher than those in rhizospheric soil, among which the Shannon and Phylogenetic diversity had significantly higher values (Mann–Whitney *U* tests, *P* < 0.05; Figures 5A,G). Specifically, the Shannon diversity and Heip’s evenness of bacterial communities in the bulk soil was only significantly higher than those in rhizospheric soil for *H. altissima* (Mann–Whitney *U* tests, *P* < 0.05; Figures 5A,E), and the Chao1 richness and Phylogenetic diversity of bulk soil only showed significantly higher values than those in the rhizospheric soil for *P. arundinacea* (Mann–Whitney *U* tests, *P* < 0.05; Figures 5C,G). In addition, the Shannon diversity, Heip’s evenness, and Phylogenetic diversity of the bacterial communities generally did not show significant differences between rhizospheric soil of different plant species (Mann–Whitney *U* tests, *P* > 0.05; Figures 5A,E,G). However, the Chao1 richness of the bacterial community in the rhizospheric soil of *P. arundinacea* was significantly lower than that of *P. communis*, *H. altissima*, and *T. sacchariflora* (Mann–Whitney *U* tests, *P* < 0.05; Figure 5C).

The spatial differences in alpha diversity of the MCB were mainly reflected in the Chao1 richness and Phylogenetic diversity (Figure 5). Both the Chao1 richness and Phylogenetic diversity in the head and central MCB were significantly higher than those in the tail (Mann–Whitney *U* tests, *P* < 0.05; Figures 5D,H). However, no significant difference was observed neither in Shannon diversity nor Heip’s evenness among the different parts of the MCB (Mann–Whitney *U* tests, *P* > 0.05; Figures 5B,F).

### Interdomain ecological networks between plants and bacteria

**FIGURE 6:**
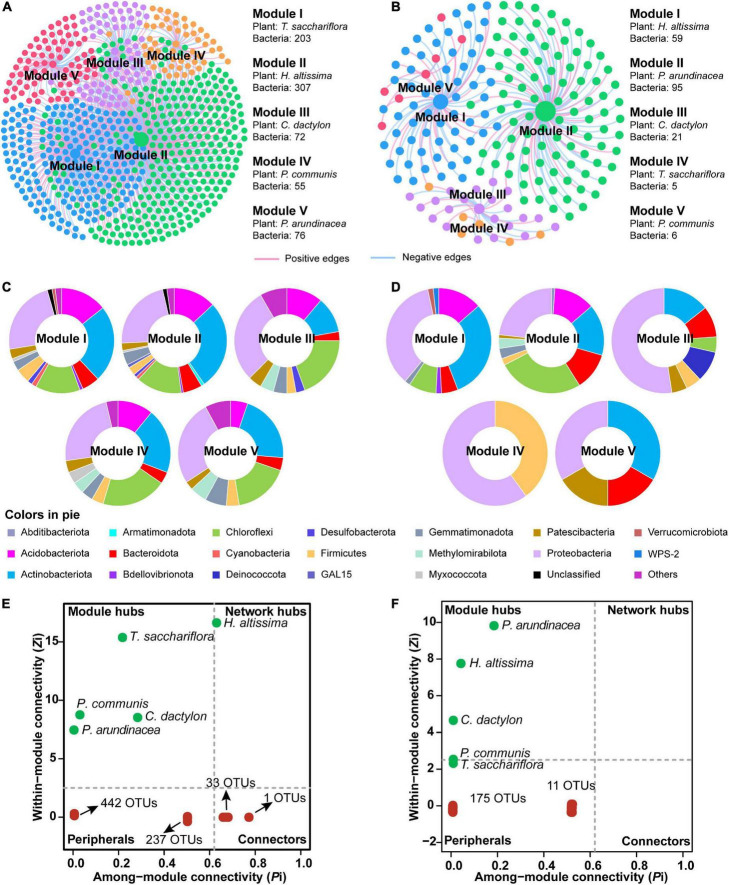
Module separation results **(A,B)**, brief summary of plant species and bacterial phyla within each module **(C,D)**, and the modular roles of plant species and bacteria based on *Zi*-*Pi* plot for the observed interdomain ecological network (IDEN) **(E,F)** between plants and bulk and rhizospheric soils, repsectively. For each module, the number of connected bacterial nodes to each plant is shown. The pie charts show the composition of bacterial operational taxonomic units (OTUs) on phylum level within corresponding modules. The relative abundances of top four phyla in each pie chart are given.

To discern the associations between plants and microorganisms, interdomain ecological network (IDEN) patterns were carried out (Figures 6A,B). The plant-microbe network for bulk soil consisted of 5 plants and 713 bacterial OTUs by 1,019 observed edges (associations) with 1.4 edges per species. The rhizospheric plant-microbe network consisted of 5 plants and 186 bacterial OTUs by 197 observed edges (associations) with 1.0 edges per species (Table 1). The connectance for bulk and rhizospheric IDENs was 0.286 and 0.212, indicating that 28.6 and 21.2% of possible edges were observed as plant-microbe associations, respectively (Table 1). Moreover, the nestedness and weighted nestedness for bulk and rhizospheric IDENs were 30.7 and 0.628, 33.9 and 0.606, respectively, which demonstrate highly chaotic structures (Table 1). In addition, the modularity, which indicates the extent to which a network can be naturally divided, reached 0.402 and 0.568 for these two IDENs, respectively, both of which had five modules (Table 1 and Figure 6A).

**TABLE 1:** Topological properties for the IDENs between plants and microbe in bulk and rhizospheric soils.

Topological properties	Plant-microbe network	
	
	Bulk	Rhizosphere
Number of plant species	5	5
Number of microbes (OTUs)	713	186
Total edges (associations)	1,019	197
Positive edges	513	77
Negative edges	506	120
Edges per species	1.4	1.0
Connectance	0.286	0.212
Web asymmetry	−0.986	−0.948
Number of compartments	1	2
Average clustering coefficient	0.121	0.113
Nestedness	30.7	33.9
Weighted nestedness	0.628	0.606
Modularity	0.402	0.568
Number of modules	5	5

For the IDEN of bulk soil, module I–V each contained one plant (*T. sacchariflora*, *H. altissima*, *C. dactylon*, *P. communis* and *P. arundinacea*, respectively) and 203, 307, 72, 55, and 76 bacterial OTUs, respectively (Figure 6A). From modules with large nodes to modules of smaller nodes, there was a trend for decreased inter-module plant–microbe associations and increased intra-module associations, suggesting complete separation (Figure 6A). Generally, the bacterial OTUs in the five modules were mainly affiliated to Actinobacteriota, Proteobacteria, Acidobacteriota, Chloroflexi (Figure 6C). The analysis of the modular roles showed that the plant species *P. communis*, *C. dactylon*, *P. arundinacea*, *T. sacchariflora* were classified as module hubs, while *H. altissima* acted as the network hub (Figure 6E). Moreover, 34 OTUs were identified as connectors (Figure 6E) playing important roles in the network. By contrast, most of the bacterial OTUs in the network were identified as peripherals (specialists) (Figure 6E), which are less important in maintaining a network.

For the rhizosphere IDEN, five plant species (*H. altissima*, *P. arundinacea*, *C. dactylon*, *T. sacchariflora*, *P. communis*) also belonged to different modules which contained 59, 95, 21, 5, and 6 bacterial OTUs, respectively (Figure 6A). Unlike modules in bulk soil, the compositions of bacterial OTUs varies greatly among different modules in the rhizospheric IDEN (Figure 6D). Among these plant species, only *P. arundinacea*, *H. altissima*, and *C. dactylon* were identified as module hubs, while other plant species and all bacterial OTUs were classified as peripherals (Figure 6F).

### Factors determining the structure of soil bacterial community

**FIGURE 7:**
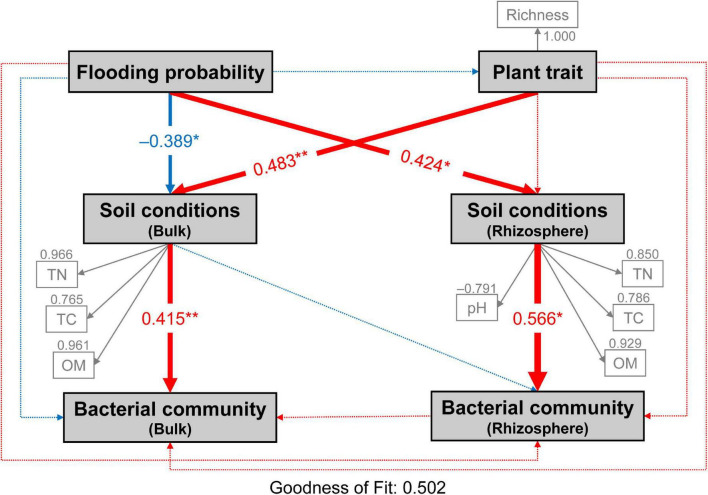
Directed graph of the partial least squares path model (PLS-PM) showing the effects of flooding disturbance and plant trait on the bacterial communities of bulk and rhizosphere soil. Flooding probability represents the flooding disturbance which is calculated as the percentage of times (days) each site was flooded in the past year until sampling day. Each box represents observed or latent variables. The observed variables with loading <0.7 were excluded in the PLS-PM construction. Larger path coefficients are reflected in the width of the arrow with red indicating a positive effect and blue indicating a negative effect. Path coefficients differ significantly from zero are indicated by “*” or “**” at 0.05 or 0.01 significance level based on 1,000 resampled bootstraps. TN, total nitrogen; TC, total carbon; OM, organic matter; Richness, plant richness.

Partial least squares path model (PLS-PM) analysis was used to assess the direct and indirect effects of flooding disturbance, plant trait and soil conditions on both bulk and rhizospheric soil bacterial communities (Figure 7). Flooding disturbance had significantly negative (–0.389) and positive effects (0.424) on bulk and rhizospheric soil conditions, respectively (bootstraps, *P* < 0.05; Figure 7). Meanwhile, plant community trait (0.483) showed a large direct effect on bulk soil conditions. The bacterial community in bulk and rhizospheric soil were significantly affected by soil conditions (bulk soil: 0.415; TN, TC, OM and rhizospheric soil: 0.566; TN, TC, OM, pH, respectively) (Bootstrap, *P* < 0.01; Figure 7). Both flooding disturbance and plant community trait had no direct effect on the bacterial community in neither bulk nor rhizospheric soil (bootstraps, *P* > 0.05; Figure 7). However, flooding disturbance and plant trait showed indirect effects on the bulk bacterial community through bulk soil conditions (−0.161 and 0.200, respectively). In addition, flooding disturbance had positive indirect effects (0.240) on rhizospheric soil bacterial community through soil conditions of the rhizosphere (Figure 7).

## Discussion

### Spatial distribution of soil nutrients and plant communities

In this study, the relatively higher TC and TN contents of bulk soils in the central part of the MCB than in either end was closely related to frequency of water flooding (Spearman correlation, *P* < 0.05; Supplementary Table 3). Soil in the central part of the MCB (quadrants B1, 2, 3 and C1, 2, 3) experienced less erosion by water flow, and thus had a more stable condition allowing for the accumulation of nutrients and thereby plant growth. High content of soil nutrients can promote the colonization and growth of plants (Sardans et al., 2017; Muñoz et al., 2021), which is consistent with the higher diversity and richness of plant communities in the central MCB found in the present study. Plant growth also promotes the accumulation of soil nutrients through rhizodeposition, root exudates and litter inputs (Cotrufo et al., 2015; Guo et al., 2019; Villarino et al., 2021). As a contrast to the central MCB, the tail zone of the MCB has recently been formed through sediment transport and deposition by the flow of the river, and which is therefore subjected to strong hydraulic disturbance (Ashworth et al., 2000; Chen et al., 2020; Wen et al., 2020). Similarly, the lower coverage and richness of plants suggested that the establishment of plant communities in the tail part was more nascent than those in the central part, which therefore resulted in lower accumulation of soil OM, TC, and TN contents. It was noted that quadrants D2 and D3, located in the central of the MCB, both had relatively low nutrient contents despite being in an area of otherwise high nutrient levels. This was probably due to their low elevation compared to other quadrants in the same part of the MCB (Figure 2).

The low-lying area had more extensive water exchange with the river through soil penetration, thereby impeding accumulation of nutrient materials. The PLS-PM suggested that flooding frequency and plant communities co-determined the soil physico-chemical conditions, and the plant communities appeared to be of greater importance with higher path coefficient. Overall, the spatial distribution of soil nutrients and plant communities suggested that the central part of the MCB was sustaining late succession whilst the head part demonstrated a less mature succession stage. The newly formed tail part sustained the most nascent habitat throughout the MCB.

### Community structure of soil bacteria

The dominance of *Pseudarthrobacter* in both the rhizospheric and bulk soils can be attributed to it being particularly well adapted to barren soils (Delgado-Baquerizo et al., 2017; Cao et al., 2021). Indeed, their spores can remain dormant through adverse conditions, facilitating their resilience to environmental stress (Andrew et al., 2012; Berendsen et al., 2016). It was evident, however, that *Pseudarthrobacter* was enriched in the head and central zones of the MCB in respect to the tail. These results suggested that although *Pseudarthrobacter* possesses unique adaptability to adverse habitats, it was inclined to thrive in nutritive and stable habitats (Volova et al., 2017). The difference in bacterial communities between the rhizospheric and bulk soils can primarily be attributed to genera with relatively low abundance (i.e., *Marmoricola*, *Chryseobacterium*, *Exiguobacterium*, *TM7a*, *Rhizobium*, *Sphingomonas*, *Massilia*). These genera undergo symbiotic relationship with plants like *Exiguobacterium*, *Rhizobium*, and *Chryseobacterium*, and are therefore present to a much larger extent in the rhizospheric soil. They have in fact been reported to possess plant growth promoting capabilities (Alami et al., 2000; Nishioka et al., 2016; Kasana and Pandey, 2018). The bacterial community in bulk soil also showed significant spatial variations. The genera of *Chryseobacterium*, *Exiguobacterium*, *Brevundimonas* and *Ellin6055* were more likely to be present in the tail zone, suggesting their competitive advantage in a newly born and less nutritive habitat. Indeed, this is consistent with the PLS-PM analysis which indicates a strong coefficient (0.415) between the composition of the bacterial community and the soil nutrients, including TN, TC, and OM.

Phylogenetic diversity considers the phylogenetic distance between species in a community, and therefore their relatedness (Srivastava et al., 2012). While the Chao1 index presents species richness and is sensitive to changes in the number of rare species in the sample (Chao, 1984). The low phylogenetic diversity found in the tail part of the MCB indicated that the species diversity and community size were smaller due to the low level of soil nutrients concurrently found in this part of the MCB. The lower Chao1 richness observed in the tail suggested that rare species of bacteria might be filtered out by the stronger disturbances in this zone, probably due to the low nutrients level or higher pH which have pervasive effects on soil bacterial diversity (Zhalnina et al., 2015; Ma et al., 2016). However, the strong water erosion in the tail zone might also play a key role in determining the microbial diversity in an MCB (Srivastava et al., 2012).

### Associations between plant and microbe

A tighter association between plant and microbial communities in bulk soil than in rhizospheric soil was confirmed by the interdomain ecological network (IDEN) analysis. However, these results differ from published concepts about the rhizospheric soil being shaped primarily by plant root material with strong microbial associations (Raaijmakers et al., 2009; Jin et al., 2022). Our results may be caused by the bulk soil containing a much higher number of total bacterial species than rhizospheric soil, thus resulting in more correlations statistically. Additionally, the aboveground community traits of plants collected in this study may not directly impact the rhizosphere soil. In bulk soil, *T. sacchariflora* (203 edges) and *H. altissima* (307 edges) were the two plant species portraying the most associations with soil bacteria by a great amount. This therefore depicts a central MCB with a more extensive plant-microbe association since the central zone is where *T. sacchariflora* and *H. altissima* were most abundant. The higher levels of soil nutrients and the stable environment found in the central part of the MCB were suitable for both plant and microbial communities, and supported extensive associations in the soil. Moreover, *H. altissima* was identified as a network hub in the IDEN, characterizing *H. altissima* as a significant plant species coordinating the establishment and development of the soil microbial community. Furthermore, the plants of *P. arundinacea* and their associated bacterial phyla Chloroflexi, Proteobacteria, Actinobacteriata and Acidobacteriota were observed primarily in the tail zone of the MCB. *P. arundinacea* is believed to be an invasive species that grows under a wide range of environmental conditions and prefers to colonize post-disturbance sites high in moisture (Galatowitsch et al., 1999; Green and Galatowitsch, 2002). The combination of *P. arundinacea* and its associated microbes may thus act as pioneers in a newly born and highly disturbed habitat like that of the tail zone of an MCB.

Typically, plants serve to refine the microbial community in rhizospheric soil. Microbial adaptions that act in harmony with specific plant traits may often result in beneficial plant-microbe associations (Bulgarelli et al., 2013). However, the plant-microbe networks of both the bulk and rhizospheric soil were characterized by a chaotic structure (non-nested structure), which contrasts previous observations in which a nested structure was found (Fortuna et al., 2010; Toju et al., 2014). Such chaotic structure of the plant-microbe networks suggested that despite a strong association between plants and microbes, each plant species did not have a role in refining the bacterial species. Indeed, this was confirmed by the results of the PLS-PM analysis which also portrayed an insignificant relationship between plant trait and bacterial communities. On the contrary, a significant coefficient between plant trait and soil condition was observed. It is assumed that measured aboveground plant trait did not have direct dominant effects on soil microbes. As such, the growing plant community could promote the establishment of plant-microbe associations by modifying soil conditions. While the plant species themselves did not directly determine the bacterial community on the MCB. It is noteworthy that different conclusions may be drawn if plant traits measured in this study are underground traits instead aboveground traits, such as underground biomass, root surface area, etc.

## Conclusions

An environmental gradient was observed in the MCB ranging from the newly-formed tail zone to the more mature head and central zones. This was evidenced by the higher plant richness and soil nutrient content in the more mature areas of the MCB. The soil microbial community was largely correlated with soil nutrients such as TN, TC and OM, suggesting the importance of maintaining healthy soil conditions to facilitate new habitat formation. For this, the regulation of water flow is essential. The plant-microbe combination between the plant *P. arundinacea* and the bacterial phyla Chloroflexi, Proteobacteria, Actinobacteriata and Acidobacteriota may act as pioneers in a newly formed habitat even though *P. arundinacea* is often regarded as an invading plant species. It should be highlighted that the effect of aboveground plant traits, such as plant richness, on microbes are mostly indirect; the plants refined the soil conditions which subsequently shaped the structure of the microbial community in the soil. Overall, this study provides valuable insights into understanding how environmental disturbance and plants cooperate in the succession of microbial community, as well as guiding the conservation of newly formed ecosystems.

## Data availability statement

The datasets presented in this study can be found in online repositories. The names of the repository/repositories and accession number(s) can be found below: https://bigd.big.ac.cn/gsa/, CRA004701.

## Author contributions

YW, FY, and XY designed the study. FY and XY performed the field work and laboratory work. FY, YW, YH, and JW analyzed the data. FY and YW wrote the manuscript. HO, SM, and TV contributed to the writing. All authors have reviewed the manuscript.

## Footnotes

1
http://www.Majorbio.com

2
http://drive5.com/uparse/

3
https://www.arb-silva.de/documentation/release-132/

4
http://huttenhower.sph.harvard.edu/galaxy

5
http://mem.rcees.ac.cn:8081

6
https://bigd.big.ac.cn/gsa/
