# Supplementary material for: Succession of soil microbial community in a developing mid-channel bar: The role of environmental disturbance and plant community
Source: Front Microbiol. 2022 Aug 17;13:970529. doi: 10.3389/fmicb.2022.970529 (PMC9428583; doi:10.3389/fmicb.2022.970529)
Supplement: Supplementary file 1 [file Data_Sheet_1.PDF]

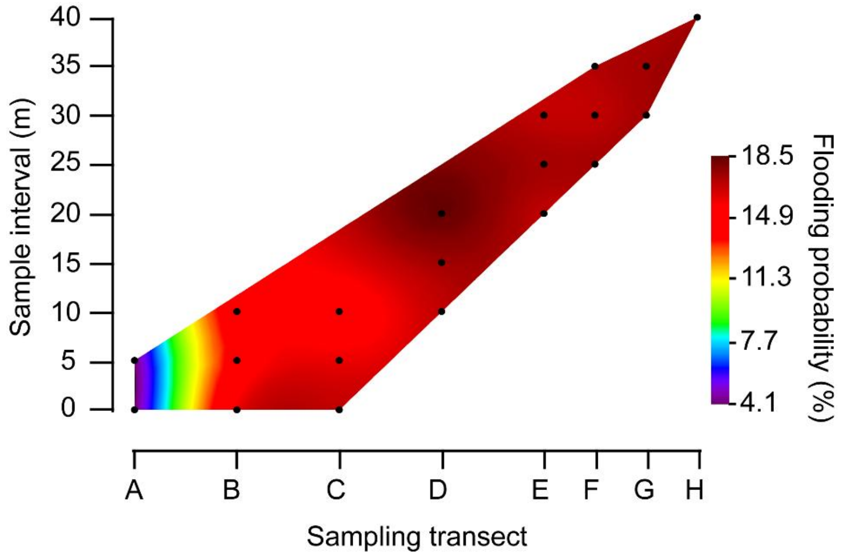

Supplementary Figure S1. Contour plots showed flooding probability of each site in the MCB. Flooding probability indicates the percentage of times (days) each site was flooded in the past year until sampling day. The black dots indicate the sampling quadrats, which correspond to the quadrat orders depicted in Fig. 1. The color fill plot represents a 3-dimensional bulk by plotting contours on a 2-dimensional format using Origin soft. Capital letters: longitudinal position of the quadrats.

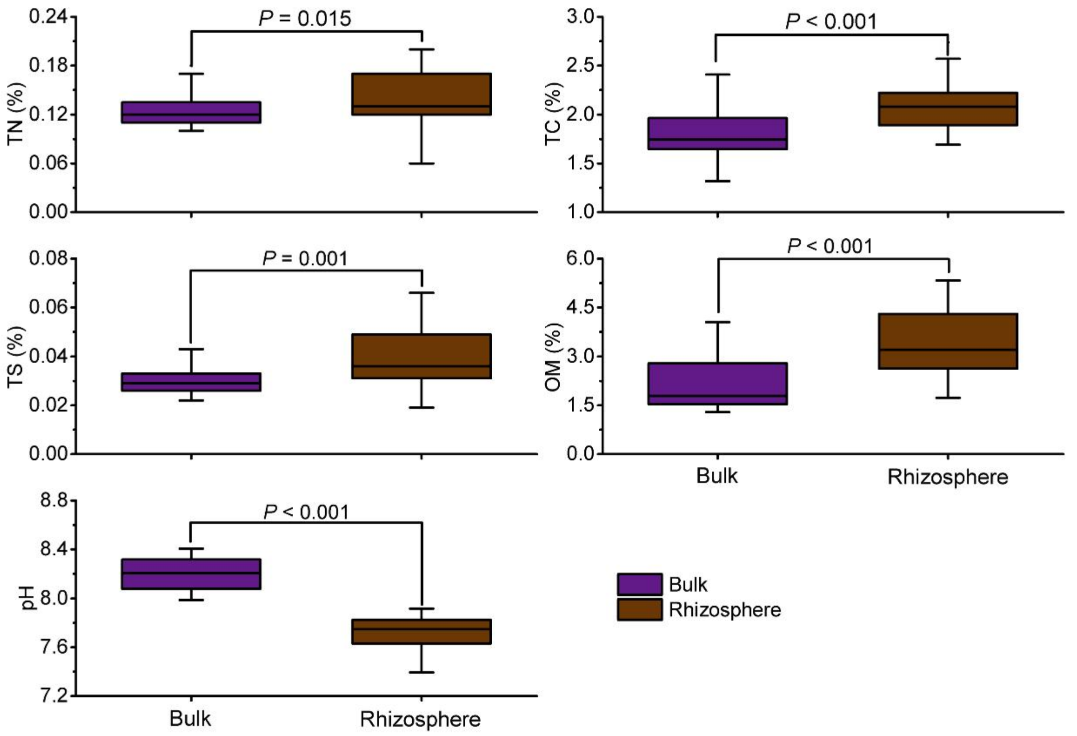

Supplementary Figure S2. Soil properties in the surface and rhizospheric soils. The *P* values with significant differences at 0.05 level based on Mann-Whitney *U* tests.

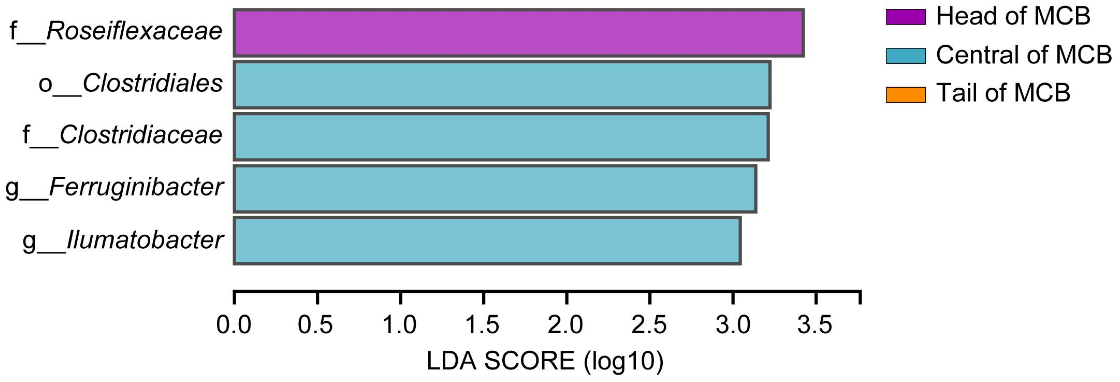

Supplementary Figure S3. Linear discriminant analysis effect size (LEfSe) of soil bacterial communities from order to genus in the head, central and tail of MCB. Linear discriminant analysis (LDA) scores larger than 3.0 are displayed. The lowercase before "__" indicates the taxonomic levels, thereinto, "o__", "f__", "g__" refer to order, family, genus, respectively.
