# Supplementary material for: Succession of soil microbial community in a developing mid-channel bar: The role of environmental disturbance and plant community
Source: Front Microbiol. 2022 Aug 17;13:970529. doi: 10.3389/fmicb.2022.970529 (PMC9428583; doi:10.3389/fmicb.2022.970529)
Supplement: Supplementary file 2 [file Data_Sheet_2.PDF]

Supplementary Table S1 Physico-chemical properties of rhizospheric soil of different plant species in each sampling quadrat.

Plant species	Quadrat	Physico-chemical properties of rhizospheric soil				
		TN (%)	TC (%)	TS (%)	OM (%)	pH
P. communis	A1	0.12	1.9	0.05	1.7	7.9
	B1	0.19	2.6	0.04	5.3	7.8
	B2	0.13	2.0	0.05	2.1	7.9
	B3	0.13	2.0	0.04	3.1	7.7
	C3	0.18	2.2	0.03	3.8	7.7
C. dactylon	A2	0.13	1.8	0.03	1.8	7.9
	B2	0.18	2.0	0.02	4.5	7.7
	E2	0.12	2.2	0.03	3.4	7.8
	G1	0.13	2.1	0.04	2.7	7.8
P. arundinacea	B1	0.20	2.4	0.05	4.9	7.6
	B3	0.06	1.8	0.02	1.9	7.8
	C1	0.13	1.8	0.04	2.6	7.8
	C2	0.17	2.1	0.06	3.2	7.6
	D1	0.12	2.2	0.03	3.0	7.8
	E1	0.10	1.9	0.03	2.9	7.9
	E3	0.10	1.9	0.03	2.9	7.9
	F1	0.13	1.7	0.03	2.9	7.8
	F2	0.13	2.2	0.05	2.6	7.7
	F3	0.13	2.2	0.04	2.7	7.8
	G2	0.13	2.3	0.04	2.4	7.8
	H1	0.11	2.0	0.05	2.2	7.8
H. altissima	B1	0.19	2.1	0.04	4.3	7.6
	B3	0.15	2.2	0.02	4.4	7.8
	C1	0.16	1.9	0.03	3.6	7.7
	C2	0.16	1.9	0.04	3.7	7.4

	D1	0.08	2.0	0.06	3.2	7.7
	D2	0.17	2.1	0.06	4.4	7.7
	D3	0.18	2.7	0.04	4.3	7.6
	C3	0.20	2.1	0.03	3.8	7.6
T. sacchariflora	D2	0.17	2.4	0.07	4.5	7.6
	D3	0.17	2.4	0.04	4.6	7.6

Note: combinations of capital letter and number: quadrat ID; capital letters and numbers: longitudinal position and transect order of quadrats, respectively.

Supplementary Table S2 Characteristics of the plant community in each sampling quadrat.

Plant species	Quadrat	Plant traits			
		Height (cm)	Coverage (%)	Richness	Importance value
P. communis	A1	190	25.6	6	97.2
	B1	140	18.0	9	15.3
	B2	220	18.4	12	41.6
	B3	160	1.9	20	1.9
	C3	120	2.6	9	2.1
C. dactylon	A2	15	1.1	6	0.3
	B2	23	67.2	12	15.9
	E2	9	0.6	9	0.3
	G1	10	9.9	2	41.5
P. arundinacea	B1	120	30.0	9	21.9
	B3	130	66.0	20	52.8
	C1	120	6.2	10	8.3
	C2	108	0.6	8	0.1
	D1	110	34.0	7	44.3
	E1	76	42.0	6	97.7
	E3	96	21.4	9	94.6
	F1	87	6.4	2	99.3
	F2	87	4.0	5	83.0
	F3	81	2.5	2	32.7
	G2	80	2.0	2	58.5
	H1	78	2.2	2	5.1
H. altissima	B1	85	72.0	9	37.2
	B3	75	34.0	20	15.7
	C1	84	60.0	10	56.8

	C2	100	2.8	9	1.9
	D1	73	56.0	7	48.4
	D2	95	72.0	18	27.8
	D3	70	11.7	16	25.6
	C3	450	80.0	8	83.2
T. sacchariflora	D2	350	32.0	18	45.5
	D3	330	28.0	16	43.8

Note: combinations of capital letter and number: quadrat ID; capital letters and numbers: longitudinal position and transect order of quadrats, respectively.

Supplementary Table S3 Spearman correlations between physico-chemical properties of bulk soils and flooding probability of sampling quadrats in the MCB.

	Spearman correlation	TN	TC	TS	OM	pH
Flooding	*r*	–0.687	–0.478	–0.505	–0.302	–0.151
probability	*P*	0.001	0.033	0.023	0.195	0.524

Note: flooding probability indicates the percentage of times (days) each site was flooded in the past year until sampling day.
